# Female Youth Soccer Participation and Continued Engagement: Associations With Community Size, Community Density, and Relative Age

**DOI:** 10.3389/fspor.2020.552597

**Published:** 2020-09-18

**Authors:** Kristy L. Smith, Patricia L. Weir

**Affiliations:** Motor Behaviour and Lifespan Development, Department of Kinesiology, University of Windsor, Windsor, ON, Canada

**Keywords:** community size, community density, birthplace, relative age, youth sport, sport participation, athlete development, environment

## Abstract

Environmental context can impact youth engagement in sport and athlete development. Previous work has examined the population size of the birthplace of elite athletes; commonly known as the *birthplace* or *community size effect*. Community density has also been recognized as an important variable. Exact estimates for the ideal community characteristics and a thorough understanding of the underlying mechanisms has been somewhat elusive. Existing studies are cross-sectional in nature and there is evidence to suggest that significant variation exists within imposed categories. An athlete's birthdate position in a similar-age cohort can also impact development and has been associated with (dis)advantages resulting from subtle age differences (i.e., the relative age effect); it remains unknown if this variable is associated with population density. The objective of this study was to establish longitudinal participation trends among female youth soccer players in Ontario Canada, with consideration of community size, community density, and relative age. Within-category variation and associations between the variables were assessed. Registration entries at age 10 years (*n* = 9,826) and 16 years (*n* = 2,305) were isolated for analysis. Odds ratio analyses were conducted within each community size and density category for all 10 year old registrants; 95% confidence intervals were obtained. This procedure was repeated for all registrants at 16 years of age using the expected distribution at age 10 years to examine continued engagement. Findings suggest medium-sized communities (i.e., 10,000–249,999 inhabitants) provide the best odds of participation and continued engagement. Less densely populated communities (i.e., 50–<400 population/km^2^) appeared to be ideal for facilitating participation at age 10 years, but not for engagement at age 16 years. However, within-category variation was evident when each community was inspected individually. Consistent with previous attempts to find an association between community size and the relative age effect, there did not appear to be an association between community density and birth quartile distribution. Observations from this study show that community size and community density are truly unique and separate variables. Future studies should consider the underlying contributions to both low and high participation and continued engagement, while being mindful of within-category variation.

## Introduction

Athletic development pathways are multifaceted and successful achievement of elite status is difficult to predict. A variety of direct (e.g., genetics) and indirect factors (e.g., opportunities for skilled instruction, location) can interact to enhance or constrain athletic potential (see Baker and Horton, 2004 for a review). Environmental context is one consideration when examining factors that can impact youth engagement in sport and athlete development, and consequently “where” an athlete is born has been recognized as an indirect contributor to athlete development (Côté et al., 2006). This “community size (CS) effect” (Baker et al., 2014) has traditionally focused on the birthplace size of professional athletes, using the location of birth as a proxy for early athlete development. For instance, Curtis and Birch (1987) produced one of the first studies to suggest the potential existence of a birthplace effect in professional and Olympic hockey players from Canada and the U.S.A. They found that the largest cities (>500,000) and rural communities (<1,000) were underrepresented as birthplaces of elite ice hockey players. Similar observations were reported for elite ice hockey players in North America (Côté et al., 2006) as well as other sports at the professional level, including basketball (Côté et al., 2006), baseball (Côté et al., 2006), football (MacDonald et al., 2009a), and golf (Côté et al., 2006; MacDonald et al., 2009b). However, the consistency of these estimates may be somewhat misleading as significant heterogeneity within similar-sized communities has been reported among Canadian National Hockey League draftees from different regions (Wattie et al., 2018; Farah et al., 2019).

Results in other parts of the world have also suggested inconsistency for CS effects; even when cultural context is controlled. For example, Baker et al. (2009b) examined the German first leagues of four sports: soccer, basketball, handball, and volleyball. While there was some evidence that communities with very small or very large populations were less likely to produce elite athletes, exceptions also occurred across the sport contexts examined. Similarly, Lidor et al. found variable “birthplace effects” among male (Lidor et al., 2010) and female athletes (Lidor et al., 2014) from several “Division I” sports in Israel. No consistent trends were identified between the two samples in any sport or population category, with the exception of elite volleyball players originating from very small communities (<2,000). Wattie et al. (2018) suggest that the inconsistencies may be attributable to broader social, political, and cultural factors both between and within countries.

The impact of CS may lie in the availability of early sport experiences. Very small, rural communities may lack facilities (e.g., ice rinks, soccer fields) and associated resources, as well as the human capital (e.g., coaches, participants, volunteers) to sustain organized leagues. Conversely, very large cities may suffer from insufficient availability of facilities and resources, leading to competition for access among community members. Indeed, several studies have shown that small to medium-sized communities (i.e., cities or towns that provide a balance between resources and demand) provide superior opportunities for young athletes in terms of participation at developmental levels, in additional to the likelihood of becoming an elite/professional athlete (e.g., Curtis and Birch, 1987). Turnnidge et al. (2014) reported higher rates of youth ice hockey participation in smaller cities (<100,000) within Ontario, Canada (male, age 8–16 years) as compared to larger communities (>100,000). Imtiaz et al. (2014) found CS was also related to longer-term participation in the same sport context in the only existing longitudinal study to date. Engagement rates of youth ice hockey players over a 7 year period (male, age 7–14 years) revealed a negative correlation with city size, with athletes from large cities (>500,000 inhabitants) being almost three times more likely to drop out of the sport during the examined timeframe.

To date, the aforementioned participation trends in youth sport do not explain the production of elite athletes with respect to CS. Rossing et al. (2016) found that elite football and handball athletes were generally more likely to come from communities with >30,000 inhabitants, despite a higher likelihood of participation among youth football and handball players in smaller communities (<30,000). However, additional examinations of both variables (i.e., youth participation and the associated likelihood of becoming an elite athlete) within other sports and cultural contexts are required before reliable conclusions can be made.

Recent studies have also suggested that community density (CD) should be considered along with CS; use of CD to evaluate athletic development contexts was first suggested in Baker et al. (2009b). Community density considers the number of people living within a specific unit of area, typically by square kilometer and may be a better indicator of the number of people drawing on available sport resources within a community. Hancock et al. (2018) examined the location of development by CS and CD for 4,062 elite, Portuguese volleyball players. While medium-sized cities (200,000–399,999 inhabitants) provided the best odds of reaching elite status in volleyball for both male and female athletes, the most elite “first-league” male players were found to come from less-densely populated areas. This trend was possibly facilitated by the availability and safe use of sport resources during development, and / or provision of a social structure that promoted athletic expertise (Hancock et al., 2018). No comparable findings were reported for females. Finnegan et al. (2017) also associated a greater likelihood of selection to talent development programs with a lower population density in a study of a study of elite youth Irish footballers.

Rossing et al. (2018) examined the developmental locations of elite and national football and handball players in Denmark, along with community-level youth as a comparison group. Odds ratio analyses suggested some inconsistencies in the optimal community size and density for athlete development in different sports (i.e., football vs. handball). However, a trend toward larger, more densely populated cities was found for elite (>30,000 inhabitants; >250 people/km^2^) and national (>50,000 inhabitants; ≥1,000 people/km^2^) football players, while mid-sized communities appeared to be best for the development of elite (between 30,000 and 100,000 inhabitants; 250–1,000 people/km^2^) and national (between 30,000 and 50,000 inhabitants; no optimal population density identified) handball athletes. Similarly, Farah et al. (2018) found a positive relationship between population density and National Hockey League draftees in all provincial regions of Canada; while also reporting significant heterogeneity when the origin of the same population of athletes (*n* = 1,502) was analyzed by CS (Farah et al., 2019). It has yet to be determined whether inclusion of the population density variable helps to explain the inconsistencies in CS research; however, it is evident that both overall size and CD should be included in future studies.

An additional indirect influence on athlete development is *date of birth* or more specifically, the athlete's birthdate position within a similar-age cohort. Generally, a birthdate closer to, but following an organizational cut-off date is associated with a sport advantage, and vice versa. The potential (dis)advantage resulting from these subtle age differences among peers grouped within the same cohort is known as the *relative age effect* (RAE) (Barnsley et al., 1985; Musch and Grondin, 2001; Wattie et al., 2008). The RAE is considered to be present when an over-representation of relatively older athletes is observed among the participant population of a particular sport, especially in competitive contexts where selection processes determine which athletes have opportunities to engage in competition (Cobley et al., 2009; Smith et al., 2018).

Relative age can potentially bias the athlete experience throughout the developmental years and ultimately influence attainment at the professional level (Côté et al., 2006; Bruner et al., 2011; Rossing et al., 2016). For example, the RAE can impact an athlete's exposure to sport in early childhood; studies have suggested that parents may be hesitant to register later-born, potentially smaller children in physical sports, such as soccer (Delorme et al., 2010) and ice hockey (Hancock et al., 2013; Smith and Weir, 2013), as inferred by lower registration numbers for the relatively youngest at the introductory levels of sport. The RAE is also believed to increase the likelihood that the relatively older—individuals who are more physically and psychologically mature due to greater accumulated life experience—will be selected to elite levels of sport at stages involving selection processes, where they will have access to higher quality training, coaching, and competition (Helsen et al., 1998; Musch and Grondin, 2001). This increased access to development opportunities can theoretically enhance the likelihood of reaching elite or professional status for the relatively oldest (Cobley et al., 2009); conversely, the relatively youngest may be at greater risk for negative sport experiences, leading to decreased competence and a decline in sport participation altogether (Barnsley and Thompson, 1988; Helsen et al., 1998; Delorme et al., 2010; Lemez et al., 2014).

The impact of CS has been examined in combination with relative age in several studies (e.g., Côté et al., 2006; Baker and Logan, 2007; Bruner et al., 2011; Turnnidge et al., 2014). To date, the published literature has shown no association between the two variables despite their potential influence on early sport experiences. Examinations of CD and the RAE are less common. Yet, competition for a position on a team has been identified as an important factor for the RAE to emerge (Musch and Grondin, 2001; Baker et al., 2009a) and thus, a high population density may predispose athletes in communities with greater competition for resources and playing positions (i.e., due to a high CD) to experience a greater risk of RAEs in youth sport. Preliminary evidence for the impact of CD on RAEs was reported by Finnegan et al. (2017); the strongest effect size for the RAE among the youth Irish footballers was observed in the most densely populated Irish province of Leinster. However, no test of the association between the two variables was conducted.

To further examine the impact of CS and CD in the realm of sport and associations with the RAE, the current study analyzed female youth soccer participation in the province of Ontario, Canada to establish longitudinal trends at the developmental level. Community size and density were examined at the category level to establish the likelihood of being a participant and subsequently remaining engaged in youth soccer across the pre- to post-adolescent transition years. Within-category variation and associations between CD and CS, and CD and the RAE were assessed to further expand current knowledge in this area and direct future research. In consideration of previous research findings, it was hypothesized that medium-sized communities with low-density populations would exhibit a greater likelihood of participation and continued engagement across the longitudinal period; but within-category variation would be observed similar to findings in other Canadian contexts (Wattie et al., 2018; Farah et al., 2019).

## Materials and Methods

A 1 year cohort of female^1^ soccer participants was identified and an anonymized dataset was created by the provincial-level governing body for developmental^2^ soccer, *Ontario Soccer* (*n* = 9,915). Registration entries were tracked over a 7 year period (i.e., age 10 to 16 years). Prior to analysis, the dataset was screened for inconsistent and/or missing information with respect to birth month. Each participant was coded by birth quartile based on the December 31st cut-off employed by Ontario Soccer for age groupings (i.e., Quartile 1 [Q1]: January through March; Quartile 2 [Q2]: April through June; Quartile 3 [Q3]: July through September; Quartile 4 [Q4]: October through December); consistent with previous research (e.g., Weir et al., 2010; Smith and Weir, 2013).

Longitude and latitude for each participant's home address were obtained using the *Google Maps Geocoding* platform. Missing or problematic postal codes were confirmed using alternate entries for the participant when available, or the entry was removed. Postal codes from outside of the province of Ontario were excluded (i.e., Michigan and Quebec). Community size (CS; overall number of inhabitants) and community density (CD; number of people per km^2^) were obtained at the census subdivision level^3^. Registration entries at age 10 years (*n* = 9,826) and 16 years (*n* = 2,305) of age were isolated for analysis, representing the pre- to post-adolescence transition years for this female cohort.

To compare participation rates in Ontario by CS and CD, the expected distribution (based on the population distribution within the province of Ontario) was compared to the observed distribution of female soccer players at age 10 years. The 2011 Canadian Census profile was selected because it was the closest available population count to the baseline year of participation under examination (i.e., 2010). Nine CS categories were applied; consistent with previous research (e.g., Baker and Logan, 2007; Baker et al., 2009b; Wattie et al., 2018). These categories are as follows: (1) <2,500; (2) 2,500–4,999; (3) 5,000–9,999; (4) 10,000–29,999; (5) 30,000–99,999; (6) 100,000–249,999; (7) 250,000–499,999; (8) 500,000–999,999; (9) >1,000,000.

No known breakdown could be obtained from research conducted within North America for CD; although one such breakdown was available for a European country (see Rossing et al., 2016). Thus, a categorization system was developed based on the actual densities found within Ontario and the overarching objective of providing a detailed analysis of CD within the province. The eight CD categories are as follows: (1) <50 people/km^2^; (2) 50–<200; (3) 200–<400^4^; (4) 400-<1,000; (5) 1,000–<1,500; (6) 1,500–<2,000; (7) 2,000–2,500; (8) =4,149.5 (i.e., Toronto). Odds ratio analyses were conducted for each category for all 10 year old, female registrants across the province; 95% confidence intervals were obtained and used to indicate statistical significance. This procedure was repeated for all registrants at 16 years of age using the expected distribution at age 10 years (to avoid bias) to examine continued engagement into the post-adolescent years. Finally, the procedure was applied for each individual community by CS for census subdivisions with a population >10,000^5^ to ascertain the presence or absence of within-category variation and identify “hot spots” for maintaining engagement in developmental soccer. These community-level ORs were then descriptively examined vs. community density to ascertain whether this variable might explain within-category variation (if present).

To examine the association between relative age and CD, a four (birth quartiles) by eight (CD categories) chi-square analysis^6^ was conducted using IBM SPSS Statistics 25. This procedure is consistent with Turnnidge et al. (2014).

## Results

### General Findings—Community Size (Age 10 and 16 Years)

The overarching purpose of this study was to examine the likelihood of participation in female developmental soccer with respect to community size (CS) and community density (CD) within Ontario. The odds ratios (ORs) and 95% confidence intervals (CIs) by CS category are presented in Table 1 (i.e., 2010 participation compared to the general population) and Table 2 (i.e., 2016 participation compared to the expected population from 2010). At the 10 year age mark within this female cohort, mid-sized communities with population size categories ranging from 10,000 to 249,999 were found to have a greater likelihood of participation based on the overall number of inhabitants (ORs ranging from 1.31 to 1.56); while very small (<4,999; ORs ranging from 0.47 to 0.63) and very large (>1 million; OR 0.44) were observed to have a decreased likelihood of participation.

**Table 1:** Odds ratios and 95% confidence intervals: Participation in 2010 compared to the general population in Ontario by community size (CS).

**CS category**	**Province of Ontario**		**Ontario soccer 2010**		**Odds ratio**	**95% confidence intervals**	
	**Total population**	**%**	**Total participants**	**%**		**Lower**	**Upper**
<2,500	180,952	1.41%	66	0.67%	**0.47**	0.23	0.72
2,500–4,999	219,575	1.71%	106	1.08%	**0.63**	0.44	0.82
5,000–9,999	617,113	4.80%	434	4.42%	0.92	0.82	1.01
10,000–29,999	1,424,976	11.09%	1,412	14.37%	**1.35**	1.29	1.40
30,000–99,999	1,830,277	14.24%	2,019	20.55%	**1.56**	1.51	1.61
100,000–249,999	2,366,327	18.41%	2,241	22.81%	**1.31**	1.26	1.36
250,000–499,999	956,161	7.44%	472	4.80%	**0.63**	0.54	0.72
500,000–999,999	2,640,694	20.55%	2,082	21.19%	1.04	0.99	1.09
>1,000,000	2,615,060	20.35%	994	10.12%	**0.44**	0.38	0.51
Total	12,851,135		9,826				

*Bolded text indicates a significant odds ratio*.

**Table 2:** Odds ratios and 95% confidence intervals: Participation in 2016 compared to 2010 by community size (CS).

**CS category**	**Ontario soccer 2010**		**Ontario soccer 2016**		**Odds Ratio**	**95% confidence intervals**	
	**Total participants**	**%**	**Total participants**	**%**		**Lower**	**Upper**
<2,500	66	0.67%	8	0.35%	0.52	0	1.25
2,500–4,999	106	1.08%	23	1.00%	0.92	0.47	1.38
5,000–9,999	434	4.42%	92	3.99%	0.90	0.67	1.13
10,000–29,999	1,412	14.37%	319	13.84%	0.96	0.83	1.09
30,000–99,999	2,019	20.55%	516	22.39%	**1.12**	1.01	1.22
100,000–249,999	2,241	22.81%	521	22.60%	0.99	0.88	1.09
250,000–499,999	472	4.80%	141	6.12%	**1.29**	1.10	1.48
500,000–999,999	2,082	21.19%	502	21.78%	1.04	0.93	1.14
>1,000,000	994	10.12%	183	7.94%	**0.77**	0.60	0.93
Total	9,826		2,305				

*Bolded text indicates a significant odds ratio*.

Communities ranging from 250,000 to 499,999 deviated from the general trend with low observed ORs for participation (OR 0.63). However, at age 16 years, this CS category maintained the highest likelihood of continued engagement (OR 1.29). Other community size categories were unremarkable in terms of keeping participants engaged at 16 years of age (i.e., ORs ~1), with the exception of very large communities greater than one million people (OR 0.77).

### General Findings—Community Density (Age 10 and 16 Years)

Community density revealed a slightly different pattern of association with participation and engagement into post-adolescence. The odds ratios (ORs) and 95% confidence intervals (CIs) by CD category are presented in Tables 3, 4. At age 10 years, communities with a CD of 50 to <400 population/km^2^ (i.e., two of the less densely populated categories in the analysis) appeared to be optimal for enhancing participation (ORs ~1.5), while participation in larger communities (i.e., 2,000 to <2,500 population/km^2^) and the largest community (i.e., Toronto) appeared to suffer (ORs of 0.85 and 0.44, respectively).

**Table 3:** Odds ratios and 95% confidence intervals: Participation in 2010 compared to the general population in Ontario by community density (CD).

**CD category (Population/km^**2**^)**	**Province of Ontario**		**Ontario soccer 2010**		**Odds ratio**	**95% confidence intervals**	
	**Total population**	**%**	**Total participants**	**%**		**Lower**	**Upper**
<50	1,989,273	15.48%	1,498	15.25%	0.98	0.93	1.04
50–<200	971,559	7.56%	1,097	11.16%	**1.54**	1.47	1.60
200–<400	1,661,603	12.93%	1,822	18.54%	**1.53**	1.48	1.58
400–<1,000	1,618,015	12.59%	1,267	12.89%	1.03	0.97	1.09
1,000–<1,500	1,901,533	14.80%	1,674	17.04%	**1.18**	1.13	1.23
1,500–<2,000	1,300,671	10.12%	954	9.71%	0.95	0.89	1.02
2,000–<2,500	793,421	6.17%	520	5.29%	**0.85**	0.76	0.94
4149.5	2,615,060	20.35%	994	10.12%	**0.44**	0.38	0.51
Total	12,851,135		9,826				

*Bolded text indicates a significant odds ratio*.

**Table 4:** Odds ratios and 95% confidence intervals: Participation in 2016 compared to 2010 by community density (CD).

**CD category (Population/km^**2**^)**	**Ontario soccer 2010**		**Ontario soccer 2016**		**Odds ratio**	**95% Confidence intervals**	
	**Total population**	**%**	**Total participants**	**%**		**Lower**	**Upper**
<50	1,498	15.25%	304	13.19%	**0.84**	0.71	0.98
50–<200	1,097	11.16%	240	10.41%	0.92	0.78	1.07
200–<400	1,822	18.54%	477	20.69%	**1.15**	1.04	1.26
400–<1,000	1,267	12.89%	331	14.36%	**1.13**	1.00	1.26
1,000–<1,500	1,674	17.04%	432	18.74%	**1.12**	1.01	1.24
1,500–<2,000	954	9.71%	204	8.85%	0.90	0.75	1.06
2,000–<2,500	520	5.29%	134	5.81%	1.10	0.91	1.30
4149.5	994	10.12%	183	7.94%	**0.77**	0.60	0.93
Total	9,826		2,305				

*Bolded text indicates a significant odds ratio*.

With respect to the association of CD with continued engagement at 16 years of age, communities with populations of 200 to <1,500 population/km^2^ appeared to be optimal (ORs ranging from 1.12 to 1.15); while very small (<50 population/km^2^; OR 0.84) and very large (4149.5 population/km^2^; OR 0.77) had an increased risk of sport dropout.

### Within-Category Variation by Community Size

To further investigate the impact of CS, ORs were calculated for all individual census subdivisions (i.e., “communities”) >10,000 inhabitants. Within the three categories that had greater than expected participation rates (i.e., significant ORs in communities of 10,000–249,999 people), community-level ORs varied considerably with greater-, neutral, and lower-than expected participation rates in each of the three categories. Odds ratios ranged from 0.08 to 4.03 in the 10,000–29,999 category; from 0.10 to 2.89 in the 30,000–99,999 category; and from 0.29 to 2.25 in the 100,000–249,999 category. Community density did not explain the variable ORs within CS categories (with one exception; see Table 5). As CS increased, CD became more variable within the CS category.

**Table 5:** Community density variation within community size and (lack of) correlation with the odds of participation.

**CS category**		**Descriptive statistics—community density (Population/km**^****2****^**)**			**Correlation between odds of participation and community density**
	**Number of communities**	**Mean**	**Standard deviation**	**Range**	
<2,500	247	45.58	112.33	0.01–728.32	N/A
2,500–4,999	62	37.88	138.55	0.02–865.31	N/A
5,000–9,999	85	140.66	280.26	0.02–1148.87	N/A
10,000–29,999	87	170.61	316.00	7.10–1791.62	−0.06
30,000–99,999	32	503.64	538.83	14.49–2086.30	0.08
100,000–249,999	16	1060.60	600.24	42.18–1838.04	0.02
250,000–499,999	3	1114.64	279.31	870.61–1419.28	0.14
500,000–999,999	4	1297.26	1065.85	316.60–2439.93	−0.92
>1,000,000	1	N/A	N/A	N/A	N/A

*Sample sizes for participants in communities of <10,000 inhabitants were considered to be too small within this 1-year cohort and therefore, community level ORs were not calculated and a correlation with community density is not available*.

The deviation from the general trend was explored in the 250,000–499,999 category; ORs were variable as observed in other categories (ORs ranging from 0.33 to 1.10). Yet, the community^7^ with the lowest odds of participation at the 10 year age mark (OR 0.33, 95% CIs 0.11, 0.55), also maintained the highest level of player engagement at age 16 years within this category (OR 1.97, 95% CIs 1.52, 2.42). It should also be noted that this particular category considered a lower number of communities due to the overall population distribution within Ontario, resulting in a smaller sample size vs. other community categories.

### Relative Age and Community Density

There did not appear to be any association between birth quartile (representing relative age) and CD. Results of the four by eight chi-square analysis were not statistically significant, χ^2^ (21, *n* = 9,826) = 14.876, *p* > 0.05. Thus, there was a failure to reject the null hypothesis.

## Discussion

### Overall Findings

The objective of this study was to examine the longitudinal participation trends in a female cohort of youth soccer players (age 10 to 16 years) in Ontario, Canada with consideration of community size (CS), community density (CD), and relative age (i.e., the RAE). Intra-category variation was assessed, and associations between these indirect contributors to athlete development were explored. In line with hypotheses, medium-sized CS categories ranging from 10,000 to 249,999 people were found to have the greatest likelihood of participation when compared to the population distribution in Ontario, while very small (<4,999) and very large (>1 million) had significantly lower participation than expected. Significant within-category variation was observed upon detailed examination of each respective community. The greatest likelihood of participation was associated with CD categories of 50–<400 people per square km (notably, defined as “rural” by Statistics Canada, 2017) at age 10 years; however, a shift toward increased odds of engagement in CD categories with mid-range densities was observed at age 16 years. There were no differences in birth quartile distribution with respect to CD, suggesting no association between these two variables.

### Community Size

The favorable likelihood of participation observed in medium-sized community categories are somewhat consistent with previous findings for youth sport participation in Canada. However, the ideal CS for *female soccer* players appears to be slightly larger (i.e., between 10,000 and 249,999 inhabitants) than the favorable estimates for *male ice hockey* players (i.e., categories of <99,999 people; Turnnidge et al., 2014). Medium-sized communities may experience higher participation rates for a variety of reasons including greater access to club membership and facilities compared to larger communities, which may suffer from a population to resource imbalance (Curtis and Birch, 1987). Yet, medium-sized communities are still large enough to sustain organized leagues, which may be difficult in a rural community with a small population, especially if it is in a geographically remote location. The sport environment of larger cities might also be more competitive because there are more participants to accommodate; this could lead to an emphasis on performance and winning over enjoyment and personal development, which may have a negative impact on long-term sport participation (Weiss and Williams, 2004; Cervelló et al., 2007; Hancock and Côté, 2014). These findings are also likely to be consistent with the observed advantages of residing in medium-sized communities with respect to becoming an elite athlete. Although this variable was not assessed in this analysis, the availability of a larger pool of athletes can theoretically enhance the level of competition experienced during youth and consequently, facilitate athlete development in the long-run.

The likelihood of maintaining engagement at 16 years of age was not as closely tied to medium-sized communities as the participation rates originally observed at the age of 10 years. The ideal category based on OR analysis was 250,000–499,999 (OR 1.29, 95% CIs 1.10, 1.48); notably, a category that also had a lower likelihood of participation at 10 years of age (OR 0.63, 95% CIs 0.54, 0.72). This trend could possibly suggest that membership is not particularly inclusive, or that other options exist for organized sport in the community; but those who do maintain engagement with local soccer clubs have positive experiences. The 30,000–99,999 category also appeared to be advantageous (OR 1.12, 95% CIs 1.01, 1.22) in terms of maintaining engagement; while all other mid-sized categories hovered near an OR of 1.0, indicating engagement rates were aligned with the observed participation numbers at 10 years of age. Very small (<2,500 inhabitants^8^) and very large (>1 million inhabitants) had a low likelihood of maintaining engagement at 16 years of age. This finding of a low likelihood for continued engagement in the largest category differs from a previous longitudinal study in Ontario, Canada. Specifically, Imtiaz et al. (2014) found an OR of 2.88 (95% CIs 2.52, 3.29) for the largest category included in the study (i.e., >500,000 inhabitants). Notably, this category is not directly comparable to this study which employed a high endpoint of greater than one million people, which solely represented the city of Toronto, Ontario. Further, Imtiaz et al. examined male ice hockey players who likely develop under different organizational sport structures and cultural attitudes about participation in their respective sport in Canada.

The different categories employed in this line of research also highlight a recent criticism of community size research. The use of wide population categories can potentially hide meaningful variation within the categories themselves (Wattie et al., 2018). Indeed, this was the case when engagement rates for individual communities were examined and has also been observed for National Hockey League draftees across Canada (Farah et al., 2019). Underlying reasons for these findings are likely multifactorial and variable between regions. For instance, geographic location may impact participation in communities of comparable size (e.g., adverse climates in northern regions, proximity of neighboring communities for competition purposes and associated travel time); the characteristics of the clubs themselves may influence participation and continued engagement (e.g., an emphasis on inclusion, participation, and development vs. performance and winning); and decision-making at the municipal level determines allocation of funding and consequently, the number and type of facilities and programming that are available to residents. Farah et al. (2019) also suggested that socioeconomic contributors (e.g., affecting the affordability of organized sport) and ethnic diversity (e.g., affecting cultural importance of the sport within the community) may impact athletic pursuits and achievement.

### Community Density

A high likelihood of participation was associated with less densely populated communities in Ontario at age 10 years, with the best odds of participation found in the categories of 50–<200 people/km^2^ (OR 1.54, 95% CIs 1.47, 1.60) and 200–<400 people/km^2^ (OR 1.53, 95% CIs 1.48, 1.58). Conversely, densely populated cities appeared to have a detrimental impact on participation as observed in the ORs for the 2,000–<2,500 people/km^2^ category (OR 0.85, 95% CIs 0.76, 0.94) and 4,194.5 people/km^2^ (OR 0.44, 95% CIs 0.38, 0.51). Comparisons to previous research are not available with respect to the impact of CD on participation, as studies incorporating this measure have focused on the development of elite athletes as opposed to overall participation at developmental levels. Advantages in less densely populated cities have been reported for Portuguese, male volleyball players (but not for females; Hancock et al., 2018). However, inherent differences in the sport system, geography, population distribution, and other potentially relevant factors make comparisons difficult between North American and European contexts. Specific to the Canadian context, Farah et al. (2018) reported an increased likelihood of being drafted into the National Hockey League for athletes from communities with higher population densities; but, the focus of the current study was on participation and continued engagement vs. athlete achievement.

Mid-range categories appeared to be best for maintaining engagement at age 16 years and thus, might be hypothesized to be the best environment for producing elite female soccer players as the development of expertise requires ongoing opportunities for competition and training. However, the disparities between CD categories were somewhat diminished between the ages of 10 and 16 years suggesting a reduced impact of population density; and further research is needed to support this hypothesis. These findings do support the suggested mechanisms for the “birthplace effect.” While less densely populated communities may offer greater opportunities for free play and organized participation during the earlier stages of athlete development, the benefits of this environmental context may diminish if these communities are not populated enough to provide the necessary resources for higher levels of training and skill development (e.g., sport facilities, coaches and competitors; Curtis and Birch, 1987; Côté et al., 2006). More specifically, this deviation in participation trends across the developmental years could suggest different advantages associated with different environmental contexts at different timepoints; possibly reconciling the current findings with Farah et al. (2018). This hypothesis is worthy of further investigation in future studies.

Consistent with previous attempts to find an association between CS and the RAE (Côté et al., 2006; Bruner et al., 2011; Turnnidge et al., 2014), there did not appear to be a relationship between CD and birth quartile distribution. While both CD and the RAE appear to be related to sport participation and ongoing engagement (or dropout), the relationship between the place of early development and the RAE is likely complicated and not easily isolated by the statistical methods used to date. Many variables can potentially influence athlete development (Baker and Horton, 2004) and this development does not occur in a vacuum; interactions between multiple systems of the developing individual are ongoing throughout the years of sport participation (Bronfenbrenner, 1977, 1995, 1999). For instance, the study of CS and CD falls within the “macrosystem” (i.e., cultural and social forces related to sport). However, the microsystem(s) (e.g., coach—athlete relationship), mesosystem(s) (e.g., coach—parent relationship), exosystem(s) (e.g., broader sport policies) and chronosystem(s) (e.g., change over time to personal characteristics or the environment) can all play a role in the developmental process.

### Future Directions

In general, mechanisms of CS and CD are largely unknown and represent promising avenues of investigation (Hancock et al., 2018; Wattie et al., 2018). Future research in this sample population and others should investigate the contributions to both high and low participation and ongoing engagement, such as the number of/proximity to soccer facilities and open spaces for unorganized play; distances traveled both within (i.e., between home and club locations) and between neighboring communities (i.e., for competition between elite teams); the organizational structures and philosophies of local clubs (as recommended in Fraser-Thomas et al., 2010), and proximity to elite teams (Farah et al., 2018; Rossing et al., 2018). This type of research can inform strategies to increase participation at the local level. For instance, community officials and sport administrators can utilize current and future research to promote a sporting structure that enhances the self-concept of individual athletes. Consideration of more inclusive sport systems (e.g., reducing team selections, smaller teams to increase playing time) and a focus on creating a sense of team identity (e.g., establishing community support and recognition) would likely be beneficial (Hancock and Côté, 2014). The proxies of CS and CD should be considered simultaneously in future analyses as both variables have shown an association with participation rates, ongoing engagement, and the likelihood of becoming an elite athlete. Further, observations from this study show that CS and CD are truly unique and separate variables; one does not inform the other.

### Strengths and Limitations

This study adds to current literature by providing a longitudinal analysis of female developmental soccer participation with consideration of both CS and CD. Community density has been observed to be an important variable in recent studies with respect to elite athlete development, and this study is one of the first to consider a relationship with sport participation at developmental levels. Soccer is currently the most popular sport among Canadian youth (Clark, 2008; Canadian Heritage, 2013) and thus, provided an ideal sport context for examination due to the high number of participants it attracts (sport selections made by Cobley et al., 2014 and Rossing et al., 2016 with a similar rationale) and its accessibility to the local community.

The use of postal codes, geocoding, and census subdivisions provided an objective, consistent method of coding for community location and characteristics; a limitation present, but rarely discussed in previous literature. Census subdivision is consistent with municipal funding structures that may impact sport programming and facility funding. However, it should be noted that it is still subject to limitations with respect to accounting for the proximity of neighboring communities (e.g., for competition purposes, options for club membership). Further, the proportion of youth in each community size category may vary and could potentially affect the accuracy of the calculated odds ratios. The use of home location (as opposed to club location) might be criticized for not providing an exact indicator of the community in which sport participation took place. However, participation rates at age 10 years were compared to the overall population distribution within the province, which are based on location of residence; thus, the use of club location could have introduced bias and home location was the best choice for this particular analysis. Future work will expand on trends for club location. Hometown was also used by Wattie et al. (2018); and either measure is preferable to using an athlete's birthplace, which may suffer to a greater degree from geographic movement/migration and conceal effects for small communities that lack medical facilities for childbirth (Rossing et al., 2016).

The choice of CS and CD categories may affect the direction of findings in this line of research and important variation can be lost when large ranges are used (see discussion in Wattie et al., 2018). Community size categories for this study were selected to allow for comparisons with previous research; the majority of existing studies have employed a similar breakdown (e.g., Baker and Logan, 2007; Baker et al., 2009b; Wattie et al., 2018). The limitations of using these groupings in this particular study included unbalanced sample sizes at age 10 years (i.e., only three communities were included in the “250,000–499,999” category due to the population distribution in Ontario) and a very small sample size for rural communities at age 16 years. Community density categories were selected using guidelines from Statistics Canada and the actual population distributions in Ontario. However, there are no existing studies available for comparison within North America and European categories are not appropriate to use due to significant geographical differences between countries (Baker et al., 2009b; Wattie et al., 2018). Furthermore, generalization of the findings to other regions in Canada cannot be made as the population of Ontario disproportionately contributes to the national population distribution and significant variation is present between provinces (Wattie et al., 2018).

The cohort information examined in this study was collected retrospectively. Ideally, an examination of participant engagement would be conducted during the actual development process and include both male and female athletes of various ages; however, this was not logistically feasible when seeking to obtain a provincially representative sample from the provincial organization. Initial participation was measured at age 10 years and again at age 16 years, which provided a valuable analysis of the pre- to post-adolescent transition years; but it does not tell us about participants who started playing soccer in early childhood and dropped out prior to 10 years of age.

## Conclusions

Community size and community density are both associated with female soccer participation in the province of Ontario, Canada. In general, mid-sized communities appear to provide the best odds of participation and continued engagement during the pre- to post-adolescent transition years; less densely populated communities also appear to be ideal. However, future studies should be mindful of within-category variation and region-to-region differences between communities of comparable size. Additional longitudinal examinations of youth sport participation are needed to confirm these findings and unravel the underlying mechanisms contributing to these effects.

## Data Availability Statement

The data analyzed in this study was obtained from Ontario Soccer. Access to the data is not available on ethical grounds as it contains personal information.

## Ethics Statement

The studies involving human participants were reviewed and approved by Office of the Research Ethics Board—University of Windsor. Written informed consent from the participants' legal guardian/next of kin was not required to participate in this study in accordance with the national legislation and the institutional requirements.

## Author Contributions

KS and PW designed the analysis and edited the manuscript. KS performed the statistical analyses, summarized the results, and drafted the manuscript. Both authors have read and approved the final version of the manuscript.

## Conflict of Interest

The authors declare that the research was conducted in the absence of any commercial or financial relationships that could be construed as a potential conflict of interest.

## Footnotes

^1^Female samples have been under-represented in relative age research (Cobley et al., 2009). Likewise, the existing youth participation (vs. CS) studies have examined male athletes (Imtiaz et al., 2014; Turnnidge et al., 2014). Thus, a female sample was selected to address this imbalance and document participation trends in a popular youth sport context in Ontario, Canada.

^2^The term “developmental” refers to an individual who is in the process of growth or progress in his / her athletic skill development at non-professional levels (Smith and Weir, 2013).

^3^Census subdivision (CSD) refers to a municipality (as determined by provincial/territorial legislation) or areas treated as municipal equivalents for statistical purposes (e.g., Indian reserves, Indian settlements and unorganized territories; Statistics Canada, 2016). The CSD level is also associated with funding and maintenance of recreational facilities by local municipalities out of the property tax base (personal communication with G. Morin, September 25, 2017).

^4^A density of 400 or more people/km^2^ is used as a threshold to distinguish between rural areas and population centers (Statistics Canada, 2017).

^5^Sample sizes for participants in communities of less than 10,000 inhabitants were considered to be too small within this one-year cohort and therefore, community level ORs were not calculated.

^6^A two-way chi-square is a test of association and not a test of interactions among variables.

^7^The overall population size will not be provided to maintain the confidentiality of this community.

^8^The sample size may be too small to produce an accurate estimate for this category; as evidenced by the large confidence interval (see Table 2). The OR should be interpreted with caution.
